# Comorbidity and metabolic syndrome in patients with multiple sclerosis from Asturias and Catalonia, Spain

**DOI:** 10.1186/s12883-017-0914-2

**Published:** 2017-07-17

**Authors:** Antoni Sicras-Mainar, Elena Ruíz-Beato, Ruth Navarro-Artieda, Jorge Maurino

**Affiliations:** 1 Fundación Rediss (Red de Investigación en servicios Sanitarios), Barcelona, Spain; 2 Health Economics and Outcomes Research Unit, Roche Farma S.A., Madrid, Spain; 3 Department of Medical Information, Hospital Universitari Germans Trias i Pujol, Badalona, Barcelona, Spain; 4 Medical Department, Roche Farma S.A., Madrid, Spain

**Keywords:** Comorbidity, Metabolic syndrome, Multiple sclerosis, Electronic medical records

## Abstract

**Background:**

The impact of comorbidity on multiple sclerosis (MS) is a new area of interest. Limited data on the risk factors of metabolic syndrome (MetS) is currently available. The aim of this study was to estimate the presence of comorbid conditions and MetS in a sample of adult patients with MS.

**Methods:**

A retrospective, cohort study was conducted using electronic medical records from 19 primary care centres in Catalonia and Asturias, Spain. The number of chronic diseases (diagnoses), the Charlson Comorbidity Index and the individual Case-mix Index were used to assess general comorbidity variables. MetS was defined using the National Cholesterol Education Program Adult Treatment Panel III. Patients were distributed into two groups according to the Expanded Disability Status Scale (EDSS) score: 0–3.5 and 4–10.

**Results:**

A total of 222 patients were studied (mean age = 45.5 (SD 12.5) years, 64.4% were female and 62.2% presented a diagnosis of relapsing-remitting MS). Mean EDSS score was 3.2 (SD 2.0). Depression (32.4%), dyslipidaemia (31.1%), hypertension (23.0%) and obesity (22.5%) were the most common comorbidities. Overall MetS prevalence was 31.1% (95% CI: 25.0–37.2%). Patients with an EDSS ≥ 4.0 showed a significantly higher number of comorbidities (OR=2.2; 95% CI: 1.7–3.0; p<0.001).

**Conclusion:**

MS patients had a high prevalence of MetS. Screening for comorbidity should be part of standard MS care. Further studies are necessary to confirm this association and the underlying mechanisms of MS and its comorbidities.

## Background

Multiple Sclerosis (MS) is a chronic autoimmune disease that affects the central nervous system and has a high impact on the health-related quality of life of patients, their families and society [1, 2]. It is one of the most common causes of neurological disability in young adults and its prevalence is increasing throughout Europe [3]. Different epidemiological studies suggest that the prevalence of MS in Spain is also increasing [4, 5]. A recent article from the Malaga province in Spain found a prevalence of 125 cases/100,000 inhabitants (95% CI: 102–169) [6].

Comorbidity is common in patients who suffer chronic disease; including individuals suffering from MS [7]. The association of comorbidity with health-related quality of life and disability progression has resulted in comorbidity being an area of increasing importance in MS research [2, 8, 9]. Rates of mortality and comorbidities have been shown to be higher in MS patients compared to non-MS patients. A recent observational study of the United States Department of Defence administrative claims database showed that MS patients (vs. a non-MS cohort) had an increased risk of developing a broad spectrum of comorbidity such as sepsis, ischemic stroke, suicide ideation, ulcerative colitis, and cancer (lymphoproliferative disorders and melanoma) [10]. Furthermore, overall risk of postmenopausal breast cancer was 13% higher amongst MS patients according to the Swedish Cancer Registry (HR [95% CI] = 1.13 [1.02–1.26]) [11]. Recent evidence suggests that patients with MS and ≥1 comorbidities have a two-fold increased risk of non-MS-related hospitalisation compared to patients without comorbidity [12].

The metabolic syndrome (MetS) is a global public-health challenge and a complex disorder characterised by a cluster of interconnected factors which lead to an increased risk of cardiovascular disease (CVD) and diabetes mellitus type 2 [13]. Previous research has shown that for individuals with autoimmune diseases, such as rheumatoid arthritis and systemic lupus erythematosus, the prevalence of MetS is higher than national averages [14]. However, only limited and inconsistent data on MetS risk factors exists for patients with MS [15]. Therefore, the aim of this study was to analyse the presence of comorbidity in a population of patients with MS with especial emphasis on MetS and its individual components.

## Methods

A retrospective, cohort study using electronic medical records from two regions of Spain (Catalonia and Asturias) was conducted. The study analysed patients from 19 primary care centres covering a population of 315,658 inhabitants in a predominantly industrial urban setting with a medium-low socioeconomic status. The study protocol was approved by the investigational review board of the Fundació Unió Catalana d’Hospitals (Barcelona, Spain).

The study included all outpatients who required care in 2015 and fulfilled the following criteria: age ≥ 18 years; a diagnosis of MS according to the International Classification of Primary Care (IPC-2, code N86) and the International Statistical Classification of Diseases (ICD-9, ninth revision, code 340); inclusion in the long-term prescriptions program (with a record of daily dose, time interval and duration of each treatment administered); and a guaranteed regular patient follow-up (presenting ≥2 healthcare records in the computer system) [16, 17]. McDonald 2010 criteria were not used in the study because our healthcare database collected diagnosis following only IPC-2 and ICD-9 classifications.

### Variables and measurements instruments

The Expanded Disability Status Scale (EDSS) was used to assess disability [18]. For the purposes of this study, disability was defined as mild to moderate (EDSS score 0–3.5) or severe (4.0–10.0). The number of chronic diseases (diagnoses), the Charlson Comorbidity Index and the individual Case-mix Index (obtained from the Adjusted Clinical Groups [ACG] - a classification system based on the consumption of healthcare resources) were used to summarise general comorbidity variables for each patient [19, 20]. The ACG application provides resource utilization bands (RUBs), so each patient was included in one of the five mutually exclusive categories depending on overall morbidity (1: healthy or very low morbidity, 2: low morbidity, 3: moderate morbidity, 4: high morbidity, and 5: very high morbidity).

The clinical, biochemical and anthropometric parameters analysed were: systolic and diastolic blood pressure (mm Hg), body mass index (BMI, kg/m^2^), basal blood glucose (mg/dl), serum triglycerides (mg/dl), total cholesterol (mg/dl), high-density lipoprotein (HDL) cholesterol (mg/dl); low-density lipoprotein (LDL) cholesterol (mg/dl) and serum creatinine (mg/dl). The diagnosis of MetS was established when an individual had three or more components of the National Cholesterol Education Program Adult Treatment Panel III (NCEP-ATP III) diagnostic criteria: hypertriglyceridemia (fasting triglyceride concentration ≥ 150 mg/dl or treatment with triglyceride-lowering agents), dyslipidaemia (fasting HDL- cholesterol <40 mg/dl in males and <50 mg/dl in females), hypertension (systolic and diastolic blood pressure ≥ 130/85 mmHg or on antihypertensive medication), hyperglycaemia (fasting plasma glucose concentration of ≥110 mg/dl or on glucose-lowering drug treatment or a previous diagnosis of diabetes), and abdominal obesity (waist circumference > 102 cm in males and >88 cm in females) [21]. In this study, the waist circumference measurement was replaced by BMI; a BMI ≥ 28.8 kg/m^2^ was considered to be equivalent to abdominal adiposity [22].

### Statistical analysis

A descriptive analysis was performed for all variables of interest with mean values, standard deviation (SD) and percentages. The variables were analysed for the overall sample of valid patients and for stratified subgroups according to the EDSS scale score. The Chi-Square and Student’s t-tests compared variables by EDSS division. In logistic regression, odds ratios (OR) were adjusted for age, gender, comorbidity (Charlson Comorbidity Index, RUBs) and EDSS score. The SPSSWIN version 19 was used for all analyses. A *p* < 0.05 was considered statistically significant.

## Results

Amongst 299,875 subjects aged ≥18 years who required medical care, 225 patients presented a diagnosis of MS and 222 were analysed (Fig. 1).

Socio-demographic and clinical characteristics of the patients are shown in Table 1. The mean ± SD age was 45.5 ± 12.5 years and 64.4% were female; 62.2% of the patients presented a diagnosis of relapsing-remitting MS (RRMS). The mean ± SD EDSS score was 3.2 ± 2.0. Intramuscular interferon beta-1a (30.6%), subcutaneous interferon beta-1a (23.9%) and glatiramer acetate (18%) were the most common disease-modifying treatments administered. A total of 48 patients (21.8%) were not receiving immunomodulatory treatment.

**Table 1:** Socio-demographic characteristics of the sample

	EDSS 0–3.5 *n* = 152	EDSS 4.0–10 *n* = 70	Total *n* = 222	*p*-value
Mean age, years (SD)	42.5 (11.5)	52.2 (12.2)	45.5 (12.5)	<0.001
Gender, female, %	68.4	65.7	64.4	0.292
Mean time since diagnosis, years (SD)	10.5 (7.6)	19.8 (10.2)	13.4 (9.5)	<0.001
MS type, %				
RRMS	74.3	35.7	62.2	<0.001
SPMS	13.8	50.0	25.2	<0.001
PPMS	7.2	14.3	9.5	0.094
CIS	4.6	0.0	3.2	0.001
*Overall comorbidity,* mean (SD)				
Number of comorbidities	4.5 (2.7)	6.0 (3.3)	5.0 (3.0)	0.001
Charlson Index	0.7 (0.6)	1.0 (0.7)	0.8 (0.6)	0.005
RUBs	2.9 (0.8)	3.2 (0.7)	3.0 (0.8)	0.003
*Comorbid diagnoses*, %				
Depression	34.2	28.6	32.4	0.404
Dyslipidaemia	25.7	42.9	31.1	0.010
Hypertension	17.8	34.3	23.0	0.007
Obesity	22.4	22.9	22.5	0.935
Active smoking	16.4	12.9	15.3	0.490
Neoplasm	9.2	15.7	11.3	0.154
COPD	6.6	14.3	9.0	0.062
Diabetes mellitus	6.6	10.0	7.7	0.373
Asthma	7.9	2.9	6.3	0.151
Ischemic stroke	4.6	8.6	5.9	0.242
Alcoholism	3.9	5.7	4.5	0.555
Ischemic cardiomyopathy	2.6	8.6	4.5	0.047

*CIS* Clinically Isolated Syndrome; *COPD* Chronic Obstructive Pulmonary Disease; *EDSS* Expanded Disability Status Scale; *MS* Multiple Sclerosis; *PPMS* Primary Progressive MS; *RRMS* Relapsing-Remitting MS; *RUBs* Resource Utilization Bands; *SD* Standard Deviation; *SPMS* Secondary Progressive MS

Depression (32.4%), dyslipidaemia (31.1%), hypertension (23.0%), and obesity (22.5%) were the most frequent comorbidities (Table 1). The impact of comorbidity was significantly greater in the severe disability group than in group of mild/moderate impairment group: mean number of comorbidities (severe vs. mild/moderate: 6.0 vs. 4.5; *p* = 0.001), Charlson index (1.0 vs. 0.7; *p* = 0.005), and RUBs (3.2 vs. 2.9; *p* = 0.003) (Table 1).

Table 2 shows the prevalence of the main cardiovascular risk factors and metabolic syndrome. The overall prevalence of MetS was 31.1% (95% CI: 25.0–37.2%). No statistically significant differences in the MetS prevalence and the number of its components between patients with an EDSS score < 4.0 vs. ≥ 4.0 were found. All biochemical and anthropometric parameters were similar between groups. The overall prevalence of cardiovascular risk factors was 14.4% without statistically significant differences between patients with an EDSS score ≥ 4.0 (12.0%) and <4.0 (15.7), *p* = 0.658. Furthermore, patients with an EDSS ≥4.0 showed a significantly higher number of comorbidities (RUBs, OR = 2.2; 95% CI: 1.7–3.0; *p* < 0.001) and longer time to diagnosis (OR = 1.2; 95% CI: 1.1–1.3; *p* = 0.023).

**Table 2:** Prevalence of the main cardiovascular risk factors and metabolic syndrome

	EDSS 0–3.5 *n* = 152	EDSS 4.0–10 *n* = 70	Total *n* = 222	*p*-value
MetS prevalence, %	28.9	35.7	31.1	0.311
MetS components, %				
BMI >28.8 kg/m^2^	30.9	30.0	30.6	0.890
BP >130/85 mmHg (or treatment)	30.8	40.0	33.7	0.199
Triglycerides >150 mg/dL (or treatment)	14.5	22.9	17.1	0.123
Fasting blood glucose >110 mg/dL	11.7	10.0	11.2	0.772
HDL-c < 40 (men) or <50 (women) mg/dL	36.8	42.9	38.7	0.393
Number of components, SD	1.4 (1,3)	1.7 (1,5)	1.5 (1,4)	0.265
Total				
1	28.9	24.3	27.5	0.532
2	11.2	10.0	10.8	0.822
3	23.0	22.9	23.0	0.758
4	5.3	10.0	6.8	0.163
5	0.7	2.9	1.4	0.234
*Cardiovascular risk* factors, mean (SD)				
Systolic BP, mmHg	127.3 (14.8)	127.9 (15.9)	127.6 (15.5)	0.870
Diastolic BP, mmHg	74.5 (10.1)	76.2 (9.9)	75.4 (10.0)	0.726
BMI, kg/m^2^	25.9 (4.5)	26.1 (4.6)	26.0 (4.5)	0.949
Glucose, mg/dL	93.2 (17.6)	93 (19.1)	93.1 (18.1)	0.985
HbA1c, %	5.8	5.8	5.8	0.255
Triglycerides, mg/dL	105.7 (50.0)	115.1 (49.4)	108.7 (49.9)	0.545
Total cholesterol, mg/dL	198.4 (38.0)	202.2 (41.5)	199.6 (39.1)	0.691
LDL-C, mg/dL	119.5 (36.5)	124.1 (39.6)	121.0 (37.4)	0.458
HDL-C, mg/dL	59.2 (17.5)	60.3 (17.2)	59.5 (17.4)	0.366
Serum creatinine, mg/dL	1.1 (0.1)	1.1 (0.1)	1.1 (0.1)	0.888
High cardiovascular risk, %	12.0	15.7	14.4	0.658

*BP* Blood Pressure; *BMI* Body Mass Index; *EDSS* Expanded Disability Status Scale; *HbA1c* Glycated Haemoglobin; *HDL*-*C* high-density lipoprotein cholesterol; *LDL*-*C* low-density lipoprotein cholesterol; *MetS* Metabolic Syndrome; *SD* Standard Deviation

## Discussion

Comorbidity is associated with diagnostic delays, more severe disability at diagnosis, greater disability progression, cognitive impairment, increased healthcare use, and higher mortality [23]. A systematic review analysed 249 articles indicated that depression, anxiety, hypertension, hypercholesterolemia and chronic lung disease were five of the most prevalent comorbidities in MS patients, whereas thyroid disease and psoriasis were the most common autoimmune diseases [24].

Our analysis of 222 MS patients from two different regions of Spain provides support for the presence of comorbidities and MetS in MS patients, as well as a trend for increasing comorbidity with increasing MS disability. Depression, dyslipidaemia, hypertension, and obesity were the most frequently observed comorbidities. Furthermore, a 31.1% prevalence of MetS was found.

The ENRICA study showed a MetS prevalence of 22.7% (95% CI: 21.7–23.7%) in a sample of 11,143 adult subjects in Spain [25]. Information about MetS in MS is still very scarce [15]. However the results of our study agree with previous research. Similarly, Pinhas-Hamiel et al. found a MetS prevalence of 30% with no gender difference in a sample of 130 MS patients with significant disability (EDSS score ≥ 3) [26].

In a systematic review analysing 34 studies, Wens et al. found an increased CVD risk in MS patients compared to healthy controls [15]. Lalmohamed et al. showed a 3.5-fold increased mortality rate in MS compared with the general population, which was mainly caused by increased deaths due to CVD (2.4-fold) [27]. However, it is not clear whether this increased risk of CVD is related to obesity or changes in body composition, dyslipidaemia, hypertension or type II diabetes [15]. Many symptoms of MS such as mobility disability and fatigue could increase the prevalence of sedentary behaviour and may have considerable implications for the development of cardiovascular comorbidities [28]. In this context, physical exercise may be a feasible intervention targeting the CVD risk.

Our study has several limitations inherent to research based on population databases, such as the presence of missing values or diagnostic codification differences [29]. In addition, socioeconomic levels, lifestyle factors and concomitant medications were not evaluated as factors associated with CVD risk.

## Conclusions

In recent years there has been a big change in the management of MS. Treatment decisions are becoming more complex due to the introduction of several new disease-modifying treatments with a more diverse spectrum of risks and benefits [30]. Before starting a treatment, neurologists should carefully consider the state of the disease, its prognostic factors and comorbidities, response to previous treatments, and patient preferences [31, 32]. Comorbidity screening should be part of standard MS care [33]. The findings in this study may help to establish an expectation of comorbidities, identify high-risk patients, educate MS patients in preventive measures and facilitate decision-making in clinical practice [33, 34]. Further studies need to be undertaken in order to validate these finding, and to gain a better understanding of the underlying mechanisms of MS and its comorbidities.

## Abbreviations

ACG: Adjusted clinical groups
BMI: Body mass index
BP: Blood pressure
CIS: Clinically isolated syndrome
COPD: Chronic obstructive pulmonary disease
CVD: Cardiovascular disease
EDSS: Expanded disability status scale
HbA1c: Glycated haemoglobin
HDL: High-density lipoprotein
HR: Hazard ratio
ICD-9: International statistical classification of diseases
IPC-2: International classification of primary care
LDL: Low-density lipoprotein
MetS: Metabolic syndrome
MS: Multiple sclerosis
NCEP-ATP III: National cholesterol education program adult treatment panel III
OR: Odd ratio
PPMS: Primary progressive multiple sclerosis
RRMS: Relapsing-remitting multiple sclerosis
RUBs: Resource utilization bands
SD: Standard deviation
SPMS: Secondary progressive multiple sclerosis
